# Respiratory tract infection is the major cause of the ambulatory visits in children

**DOI:** 10.1186/1824-7288-37-43

**Published:** 2011-09-15

**Authors:** PeiFen Liao, MinSho Ku, KoHuang Lue, HaiLun Sun

**Affiliations:** 1 Department of Pediatrics, Chung Shan Medical University Hospital, N110, Section1, Chien-Kuo North Road; 402 Taichung, Taiwan; 2 School of Medicine, Chung Shan Medical University, N110, Section 1, Chien-Kuo North Road; 402 Taichung, Taiwan

**Keywords:** Health care, Children, Database, Taiwan

## Abstract

**Background:**

As children represent the future, ensuring that they receive proper health care should be a primary concern of our societies. Epidemiological research underpins the importance of effective child health care strategies, and highlights the need for accurate data collection; such surveys are currently lacking in Taiwan. In our descriptive studies, we compared the differences of the ten most common diseases in the year 2000 and 2009 among Taiwanese children.

**Methods:**

Data for a total of 174,651 and 142,200 visits under eighteen years old were collected from the National Health Insurance Research Database in year 2000 and 2009. A maximum of three outpatient diagnostic codes (the International Classification of Disease [ICD], ninth revision) could be listed for every visit. Data were categorized according to the principal diagnoses, age and different specialties of physicians.

**Results:**

Respiratory tract infection was the most common disease (58.21% to 44.77%). Teeth (4.90% to 5.16%) and eye (2.52% to 3.15%) problems were the also in the list of top ten diseases. In year 2009, the rate of allergic rhinitis was 2.87% in 7-18 years old group. Pediatricians were the first option for consultation, followed by ear, nose and throat specialists and family physicians. However, for the school age children group, the role of pediatricians with regards to children's health care showed a decrease in its importance.

**Conclusions:**

The amount of information relevant to child health care is rapidly expanding. The ten most common diseases of the present analysis may serve as baseline data for future evaluations of the changes of type of diseases among children.

## Introduction

Children are the most vulnerable in society because of their dependence and inability to communicate their needs, and thus their needs require special attention including health care. The health problems of children differ from those of adults [1,2]. As a result, pediatrics emerged as a medical specialty more than a century ago. The health care of children also is one of the major health topics in the World Health Organization.

Taiwan is a well-developed country and has unique health insurance system which covers more than ninety-five percent of all residents [3]. Under such a convenient insurance system, parents seek medical consultation for children not only with Pediatricians but also with internal medicine, family medicine and other non-pediatric physicians especially with regards to ambulatory visits. Assessment of the state of children's health must begin with a description of the incidence of illness and must continue with studies that show the changes that occur with time. On the other hand, a child's response to illness and stress varies with age [1,2]. It is important to examine morbidities before and after school age. This may show that different age groups have different concerns, resources, and needs.

The objective of this study was to obtain a clear understanding of the ambulatory visits of children in Taiwan by analyzing a large set of representative data from the national database, categorized according to the principal diagnoses, age and different physician specialties in year 2000 and 2009, respectively. The findings of the current study may help to identify issue that may become the foci of the attention and efforts of pediatric clinicians and policy makers.

## Material and Methods

### Data source

With its establishment, the National Health Insurance provided a new sampling dataset. For this study, the insurance beneficiary of every ambulatory visit served as the sampling unit. The sampling data were obtained from a random selection of approximately 0.2% of the ambulatory visits. The information in each computerized claim form included patients' personal identification number (ID), age, gender, prescription dates, medical care institutions, and the diagnoses relevant for the prescription. The personal, physicians' and medical care institutions' IDs in the database were scrambled in compliance with the Personal Electronic Data Protection Law. Self-treatment with over-the-counter medications or alternative health services was not included in the database.

### Data collection

Data were collected from all ambulatory visits for all patients younger than 18 years old during the period from January 1, 2000 to Dec 31, 2000 and January 1, 2009 to Dec 31, 2009. A maximum of three outpatient diagnosis codes (the International Classification of Disease [ICD], ninth revision) could be listed. Only the first diagnosis as the main diagnosis was included in our analysis. Due to the similarity of some disorders, the ICD code used three digits. The unit of ambulatory visit was "person visit number". For example, if there were three ambulatory visits for one person in 2000 and 2009, then three visits were counted.

The data was classified by age (under school age 0-6 y/o and school age 7-18 y/o). Visits to different physicians with different specialties were classified as Pediatricians (PED), Family physicians (FP), Internal medicine specialists (IM), or Ear, nose and throat specialists (ENT). And the probability of children visits to each specialty was analyzed.

### Statistical analysis

All analyses were performed using SAS version 8.2 for Windows (SAS Institute Inc, Cary, NC, USA). We used standard methods for analyzing contingency tables. For 2 × 2 tables, p values were based on the continuity-adjusted Pearson chi-square test. All p values of less than 0.05 indicated statistical significance.

## Result

The total number of ambulatory visits decreased from 174,651 to 142,200 in year 2000 as compared with 2009. The ratio of sex in the two separated years and different category of ages were similar (Table 1).

**Table 1:** An Analysis of 2000 and 2009 ambulatory visits of children

	2000		2009	
	
	Number of visit	Percentage	Number of visit	Percentage
0-6 Y/O	93210	53.4	62703	44.1
7-18 Y/O	81441	46.6	79497	55.9
Total	174651		142200	
Male	92386	52.9	75689	53.2
Female	82005	47.0	66497	46.8
Missing	260	0.1	14	0.0

In the all age groups of children, we found that the respiratory system disease was the most common system disease (63.87 to 50.90%) of children ambulatory visits in year 2000 & 2009. The second most common system disease was the digestive system disease (12.33 to 14.17%). And the third most common system disease was the neurology system disease (6.31 to 8.40%) (Table 2).

**Table 2:** Ten Common Diseases of Children Ambulatory Visits in Year 2000 and 2009

System Disease	ICD code	2000(%)	2009(%)	p	OR (95% CI)
Respiratory	460-519	63.87	50.90	0.00	0.58 (0.57-0.59)
Digestive	520-579	12.33	14.17	0.00	1.17 (1.01-0.19)
Neurology	320-389	6.31	8.40	0.00	1.36 (1.32-1.39)
Dermatology	680-709	4.13	7.06	0.00	1.76 (1.70-1.81)
Injury and Poisoning	800-999	3.36	5.22	0.00	1.58 (1.53-1.64)
Ill-defined	780-799	2.70	3.48	0.00	1.29 (1.24-1.35)
Infectious	001-139	2.64	2.57	0.22	0.97 (0.93-1.01)
Genitourinary	580-629	1.02	1.45	0.00	1.42 (1.34-1.52)
Musculoskeletal	710-739	0.77	1.43	0.00	1.86 (1.74-2.00)
Health check	V202	0.54	1.10	0.00	2.04 (1.88-2.22)

001~139: Infectious And Parasitic Diseases

320~389: Diseases Of The Nervous System And Sense Organs

460~519: Diseases Of The Respiratory System

520~579: Diseases Of The Digestive System

580~629: Diseases Of The Genitourinary System

680~709: Diseases Of The Skin And Subcutaneous Tissue

710~739: Diseases Of The Musculoskeletal System And Connective Tissue

780~799: Symptoms, Signs, And Ill-Defined Conditions

800~999: Injury And Poisoning

V202: Health supervision of routine infant or child health check

In all age groups of children, the first most common diagnosis in our study was acute respiratory infection (ICD 465) not only in the year 2000(30.15%) but also in 2009(16.22%). The second most common diagnosis was acute bronchitis and bronchiolitis (ICD 466, 9.51%) in the under six year old group, but in the 7-18 year old group was disease of the hard tissue of the teeth (ICD 521, 7.40%); the findings were the same for 2000 and 2009. The third most common diagnosis was acute nasopharyngitis (ICD 460, 7.09%) and acute sinusitis (ICD 461, 7.70%) in 2000 and 2009 for the under six year old group. However, eye disorders of refraction and accommodation (ICD 367, 5.06%) was the third most common diagnosis in the 7-18 year old group in 2009.

If we observe the ten most common diagnoses, respiratory tract infections both in the upper and lower airways (ICD 46X) were more than half of diseases noted. Disorder of teeth and disorder of conjunctiva (ICD 521, 372) were the only two non-respiratory tract infection diseases in 2000. But in 2009, disorders of refraction and accommodation, and allergic rhinitis (ICD 367, 477) were added, especially in the 7-18 year old group. Healthy child visit for physical check up (V20, 3.4%) was also in the most ten common diseases for the under six year old group in 2009 (Table 3).

**Table 3:** Ten Common Diseases of Children Ambulatory Visits in Year 2000 and 2009

Age	0-18		0-6		7-18	
	
	Diagnosis	%	Diagnosis	%	Diagnosis	%
2000	465	30.15	465	35.32	465	24.24
	466	6.98	466	9.51	521	7.40
	460	6.42	460	7.09	460	5.64
	521	4.90	461	4.35	466	4.09
	461	3.83	464	4.23	372	3.46
	464	3.66	463	3.29	461	3.24
	463	3.16	521	2.71	463	3.02
	372	2.52	462	2.54	464	3.00
	462	2.31	558	1.79	367	2.51
	487	1.70	372	1.70	487	2.31
2009	465	16.22	465	18.50	465	14.42
	466	6.64	466	9.67	521	6.53
	461	6.10	461	7.70	367	5.06
	521	5.16	460	4.61	461	4.85
	460	4.42	463	4.43	460	4.26
	463	4.12	521	3.40	466	4.25
	367	3.15	V20	3.40	463	3.87
	786	2.51	786	2.86	477	2.97
	462	2.48	462	2.75	487	2.68
	477	2.28	464	2.36	372	2.67

367 Disorders of refraction and accommodation   372 Disorders of conjunctiva

460 Acute nasopharyngitis (common cold)           461 Acute sinusitis

462 Acute pharyngitis                                          463 Acute tonsillitis

464 Acute laryngitis and tracheitis                        465 Acute upper respiratory infections of multiple or unspecified sites

466 Acute bronchitis and bronchiolitis                 477 Allergic rhinitis

487 Influenza                                                      521 Diseases of hard tissues of teeth

558 Other and unspecified noninfectious gastroenteritis and colitis

786 Symptoms involving respiratory system and other chest symptoms

For specialty care, PED was the first option when seeking medical help, ENT was second and FP was third in both 2000 and 2009. We further analyzed the different age groups and the rankings were different. ENT was the first choice for the between 7-18 year old group in 2000. PED, ENT, FP had a very similar ratio in 2009 for the between 7-18 year old group as well (Table 4).

**Table 4:** Ambulatory Visits by Specialty (percentage) for Year 2000 and 2009

	0-18ys				0-6ys				7-18ys			
	
	2000	2009	OR (95% CI)	P	2000	2009	OR (95% CI)	P	2000	2009	OR (95% CI)	P
PED	22.80	29.44	1.41 (1.39~1.43)	0.00	33.45	44.88	1.62 (1.59~1.65)	0.00	10.61	17.26	1.76 (1.70~1.80)	0.00
ENT	16.30	15.82	0.96 (0.94~0,98)	0.00	17.92	16.31	0.89 (0.87~0.92)	0.00	14.45	15.44	1.08 (1.05~1.10)	0.00
FP	8.29	14.16	1.82 (1.78~1.86)	0.00	8.22	13.92	1.80 (1.75~1.87)	0.00	8.37	14.35	1.83 (1.78~1.89)	0.00
IM	5.36	5.73	1.07 (1.04~1.10)	0.00	3.96	4.18	1.06 (1.01~1.14)	0.032	6.95	6.96	1.00 (0.96~1.04)	0.938

PED Pediatricians

ENT: Ear, nose and throat specialists

FM: Family physicians

IM: Internal medicine

## Discussion

Infection of the upper respiratory tract in children is one of the most frequent reasons people who go to see their doctor in Taiwan. Eye and teeth problems were other common diseases noted, especially in the school age group. Allergic rhinitis has also had more impact in recent years. The major health care provider was pediatricians. ENT physicians were the second option for children and the proportion of ambulatory visits by children and youth increases with increasing age.

The number of births in Taiwan has been decreasing since 1970 and is expected to continue to decrease at 1-2% annually. There were 305,312 live births in 2000 but 191,310 in 2009 in Taiwan[4]. The proportion of children is decreasing relative to the adult population. Based on our data it is reasonable that ambulatory visit volume decreased. In 2000, about 174,600 visits were made to physicians' offices by children younger than 18 years old and this decreased to 142,200 in 2009.

In our study, the principal diagnoses accounting for almost 14-35% of these visits were acute respiratory tract infection (ICD 465). Coughs, colds, sore throats and runny noses are common in the lives of children and often diagnosed as acute respiratory infection or common cold. Usually they are no cause for alarm. In some cases, coughs are maybe danger signs of more serious illnesses, such as pneumonia. Over 90% of infections of this type are caused by viruses [5]. Respiratory tract infections in children also account for 30% of absences from school, and family routines are disrupted [6]. On average, a child 4 years old or younger has more then six infections of the upper respiratory tract a year, and this frequency could double for children of this age in day care center or in the kindergarten[7]. However, more than half of children with suspected acute viral infection of the upper respiratory tract are still unwell four days after their initial consultation, and a quarter are still unwell after a week (about 10 days after the onset of the illness)[8]. Above of all, this causes parents and care-givers to be more worried about minor infections. Doctors may tell the caregivers (parents, family or guardians) that children will get better in a few days with just conservative treatment at home. Giving this information to parents may enable them to care for their child more effectively and reduce the need for additional consultations. On the other hand, all children have the right to quality health care to ensure that respiratory infections and other illnesses are accurately diagnosed and treated before they become serious health risks.

Eye problems are one of the major causes for ambulatory physical visits. Indeed, a study of survey was performed in 2000 to determine the prevalence of myopia among schoolchildren in Taiwan [9]. The myopia rate increased from 20% at 7 years, to 61% at 12 years, and 81% at 15 years. A myopic rate of 84% was found for children aged 16 to 18 years. The prevalence and severity of myopia in schoolchildren in 2000 increased compared to 1986 and the most severe increase occurring in younger age groups [10]. Such increases are reflected to our study. The diagnosis of eye disorders of refraction and accommodation (ICD 367) greatly increased in school age children in 2009. Helping to prevent school age children from developing myopia at a young age is an important policy in order to slow the increase in severity of myopia in Taiwan.

The incidence of allergic diseases has increased substantially in the last two decades throughout the world [11]. Allergic rhinitis is the most common disease among allergic disease. Especially in adolescents and children, it has become a major public-health problem in developed and developing countries [12]. A large-scale cohort study in Taiwan revealed a rising trend of allergic rhinitis, from 12% to 27.6% from 1996-2002 [13,14]. Similar results have also been reported worldwide [15-17]. Unlike asthma acute exacerbation, allergic rhinitis can not induce emergency health conditions and mortality. But from the patients' perspective, allergic rhinitis is not a minor disturbance, as it has a severe negative influence on different areas of daily activity such as difficulty in sleep, concentration, general health, performance at school, and social relationships, when compared to healthy people [18].Increased attention focusing on the treatment and prevention of allergic disease is a further direction that should be explored to improve the quality of life for these patients and their family.

Oral health disparities continue to pose critical challenges as dental caries is a common chronic disease of childhood. A current review of the available epidemiological data from many countries indicates that there is a marked increase in the prevalence of dental caries [19]. This global increase in dental caries prevalence affects children when left untreated results in pain, bacteremia, high teeth care expenditure, speech disorders, and premature tooth loss with its sequelae of compromised chewing, loss of self-esteem, and harm to the permanent dentition. In our survey, oral health especially with regards to teeth care persisted as one of the major causes of non-respiratory disease in all age groups 2000 and 2009. In the school age group, it was the second common cause of ambulatory visit with a rate of 6.53-7.4% (ICD 521 in Table 3). A return to the public health strategies that increase in dental caries signals a pending public health crisis. Otherwise, dental caries in primary teeth is a preventable and reversible infectious disease. It is essential for the promotion of optimal oral health. Primary healthcare physicians should provide anticipatory guidance and educate parents and young children after the first tooth erupts or by 12 months of age [20].

ENT specialist visits were the second popular medical consultation for children, just after pediatricians. In the school age group, the role of pediatricians for health care decreased its importance in Taiwan. The cause for ENT special visits is similar to pediatricians. Respiratory tract infection was still the major disease of concern for children in Taiwan to ENT visit (data not showed). This reflects the special medical culture and habits in Taiwan. On the other hand, pediatric care became less important in the school age group with about 10.61 to 17.26%, the role of other specialists care such as dentist or ophthalmology became more important with about 29.77 to 36.75%.

Some limitations to this study are worth noting in our survey. There were a lot of different causes for patient visits other than the ten most common diseases listed (Table 3). Other diseases are also important, but are not shown in the table due to their lower rates such as pneumonia (about 1.35% in 2000). Only analyzing the principle diagnosis was other limitation. As we stated, the maximum three diagnosis codes were listed in one ambulatory visit. We only used the first code for our study. The major cause of visits should be put on the first code as the National Health Insurance policy suggests. Otherwise, in most children, their diseases were not complicated. Our data still showed results that were based on the situations faced by children in the real world.

The shaping of health care systems to meet the needs of children and their families requires statistical data and difficult decisions in setting priorities. In Taiwan, except respiratory tract infection, eye, dental and allergic rhinitis concerns should have more rational strategies for primary and secondary prevention. Our study served as baseline data for adequately addressing the special health care needs of children. It also reflected that over time, the relative importance of the various causes of childhood morbidity may undergo major changes especial in school age children.

## Competing interests

The authors declare that they have no competing interests.

## Authors' contributions

PL carried out the study design and drafted the manuscript. MK performed the statistical analysis. KL participated in its design. HS conceived of the study, and participated in its design and coordination. All authors read and approved the final manuscript.
